# Theta-Gamma Coupling and Working Memory in Alzheimer’s Dementia and Mild Cognitive Impairment

**DOI:** 10.3389/fnagi.2018.00101

**Published:** 2018-04-16

**Authors:** Michelle S. Goodman, Sanjeev Kumar, Reza Zomorrodi, Zaid Ghazala, Amay S. M. Cheam, Mera S. Barr, Zafiris J. Daskalakis, Daniel M. Blumberger, Corinne Fischer, Alastair Flint, Linda Mah, Nathan Herrmann, Christopher R. Bowie, Benoit H. Mulsant, Tarek K. Rajji

**Affiliations:** ^1^Campbell Family Mental Health Research Institute, Centre for Addiction and Mental Health, Toronto, ON, Canada; ^2^Temerty Centre for Therapeutic Brain Intervention, Centre for Addiction and Mental Health, Toronto, ON, Canada; ^3^Geriatric Psychiatry Division, Centre for Addiction and Mental Health, Toronto, ON, Canada; ^4^Department of Psychiatry, University of Toronto, Toronto, ON, Canada; ^5^Keenan Research Centre for Biomedical Science, St. Michael’s Hospital, Toronto, ON, Canada; ^6^Centre for Mental Health, University Health Network, Toronto, ON, Canada; ^7^Rotman Research Institute, Baycrest Health Sciences Centre, Toronto, ON, Canada; ^8^Sunnybrook Health Sciences Centre, Toronto, ON, Canada

**Keywords:** mild cognitive impairment, Alzheimer’s dementia, electroencephalography, neural oscillations, theta-gamma coupling, working memory

## Abstract

Working memory deficits are common among individuals with Alzheimer’s dementia (AD) or mild cognitive impairment (MCI). Yet, little is known about the mechanisms underlying these deficits. Theta-gamma coupling—the modulation of high-frequency gamma oscillations by low-frequency theta oscillations—is a neurophysiologic process underlying working memory. We assessed the relationship between theta-gamma coupling and working memory deficits in AD and MCI. We hypothesized that: (1) individuals with AD would display the most significant working memory impairments followed by MCI and finally healthy control (HC) participants; and (2) there would be a significant association between working memory performance and theta-gamma coupling across all participants. Ninety-eight participants completed the N-back working memory task during an electroencephalography (EEG) recording: 33 with AD (mean ± SD age: 76.5 ± 6.2), 34 with MCI (mean ± SD age: 74.8 ± 5.9) and 31 HCs (mean ± SD age: 73.5 ± 5.2). AD participants performed significantly worse than control and MCI participants on the 1- and 2-back conditions. Regarding theta-gamma coupling, AD participants demonstrated the lowest level of coupling followed by the MCI and finally control participants on the 2-back condition. Finally, a linear regression analysis demonstrated that theta-gamma coupling (*β* = 0.69, *p* < 0.001) was the most significant predictor of 2-back performance. Our results provide evidence for a relationship between altered theta-gamma coupling and working memory deficits in individuals with AD and MCI. They also provide insight into a potential mechanism underlying working memory impairments in these individuals.

## Introduction

Alzheimer’s dementia (AD) is the clinical manifestation of a neurodegenerative disorder characterized by pervasive and progressive cognitive and functional impairments. Recent research towards early detection and possible prevention of AD has focused on a prodromal phase of the disease known as mild cognitive impairment (MCI; Nestor et al., 2004; Galluzzi et al., 2013). Individuals with MCI display cognitive deficits greater than what is expected for their age, however, these deficits do not yet interfere with activities of daily living (Petersen et al., 2001). The incidence of AD in the general population over 65 years old is 1%–2% per year, but is as high as 15% in individuals with MCI (Petersen et al., 1999). While converging epidemiologic (Mitchell and Shiri-Feshki, 2009), neuropsychological (Petersen et al., 1999) and neurophysiologic (Jack et al., 1999; Sabbagh et al., 2010) research has established a link between MCI and AD, the underlying mechanisms associated with the progression from MCI to AD are poorly understood.

A potential mechanism associated with this progression implicates prefrontal cortical activity. Aberrant prefrontal activity is common in individuals with AD (Perry and Hodges, 1999). These deficits involve disruptions in synaptic plasticity (van Veluw et al., 2012), neuronal loss (Reed et al., 1989; DeKosky and Scheff, 1990) and aberrant corticocortical connections (Haxby et al., 1990; Leuchter et al., 1992), which underlie impaired prefrontal function including executive functioning and working memory (Haxby et al., 1990). Individuals with MCI do not display significant deficits in prefrontal activity; however, deficits in activity within this region have been shown to predict progression to AD (Gomar et al., 2011). In spite of these insights, the neurophysiologic underpinnings of prefrontal function and the association between prefrontal activation and function in individuals with MCI and AD are not clear.

Thus, the overall goal of this study is to identify a novel neurophysiologic index of prefrontal cortical activity underlying prefrontal function in AD or MCI. The prefrontal cortex is implicated in several executive processes associated with various stages of memory formation and retrieval, particularly working memory. Working memory is defined by the ability to select, maintain and manipulate information online over short time intervals (Curtis and D’Esposito, 2003). Working memory functioning is supported by local neuronal circuits within the prefrontal cortex and re-entrant circuits connecting the prefrontal cortex to more posterior cortices (Fuster, 1997). These circuits result in neuronal oscillations which are driven by repetitive and synchronized firing of groups of neurons. These oscillations display distinctive changes in response to cognitive, motor and sensory inputs (Engel et al., 2001; Buzsáki and Draguhn, 2004). A fundamental feature of oscillatory activity is neuronal coherence. This process is observed not only across neuronal networks and brain regions but also between frequency bands (known as cross-frequency coupling; Varela et al., 2001) and is a critical component of healthy cognitive functioning (Engel et al., 2001; Uhlhaas and Singer, 2006). Studies suggest that cross-frequency coupling between the phase of theta (4–8 Hz) and amplitude of gamma (30–80 Hz; theta-gamma coupling, TGC) underlies working memory processes (Engel et al., 2001; Canolty and Knight, 2010). In particular, TGC codes for the ordering of items of information during working memory time intervals, i.e., the manipulation component of working memory (Lisman and Jensen, 2013; Rajji et al., 2017). In contrast, gamma oscillations represent these individual items of information, while theta oscillations represent the time interval during which the items are held in memory (Lisman and Jensen, 2013).

At a neurophysiological level, TGC is thought to represent a code for ordering, given that different neuronal assemblies fire at consecutive time points (Lisman and Idiart, 1995; Lisman and Buzsaki, 2008). More specifically, distinct neuronal assemblies represent individual items of information through their spatial pattern of activation. Each neuronal assembly fires during a specific gamma cycle; items that follow in the sequence are represented by consecutive gamma cycles. Given that the cycle of theta is slower than that of gamma, the assemblies that fire within each gamma cycle are associated with different phases of the theta cycle (Lisman and Buzsaki, 2008). Furthermore, the activation of distinctive neuronal assemblies leads to greater differences in amplitude between subsequent gamma oscillations along a theta cycle. This ultimately results in a stronger modulation (i.e., coupling) of the amplitude of gamma by the phase of theta (Tort et al., 2010).

The current study assessed TGC and working memory in individuals with MCI and AD using the N-back task, a verbal working memory task. We assessed performance on the 1 and 2-back conditions with our primary analyses focused on the more challenging 2-back condition. The aims of this analysis were: (1) to evaluate working memory performance and frontal TGC during the N-back working memory task in AD, MCI and healthy controls (HCs); and (2) to characterize the relationship between working memory performance and frontal TGC in these groups. We hypothesized that individuals with AD would display the most significant impairments both on working memory performance and TGC followed by the MCI and finally HC groups. Additionally, we hypothesized that there would be a significant association between N-back performance and TGC across the three groups.

## Materials and Methods

### Participants

Participants were recruited through clinical referrals of individuals with MCI and AD and advertisements posted in local newspaper, magazines and hospitals. All participants provided written informed consent, as approved by the the Research Ethics Board at the Centre for Addiction and Mental Health. This article combines baseline data from two intervention studies (clinicaltrials.gov identifiers: NCT01847586 and NCT02386670). Both studies were conducted using the same equipment and N-back and electroencephalography (EEG) protocols.

Eligibility was evaluated through an initial telephone screening, followed by an in-person comprehensive clinical assessment. Eligibility criteria for AD participants included: (1) National Institute of Neurological and Communicative Disorders and Stroke and the Alzheimer’s Disease and Related Disorders Association (NINCDS-ADRDA; McKhann et al., 1984) core criteria for probable AD; (2) Structured Clinical Interview for DSM-IV–TR (American Psychiatric Association, 2000) criteria for dementia due to probable Alzheimer’s disease; (3) age of 65 years or older; (4) either not taking an acetylcholinesterase inhibitor or taking a stable dose for at least 3 months; (5) no Axis I diagnosis other than Dementia of the Alzheimer type within the past 12 months; and (6) Mini Mental State Examination (MMSE; Folstein et al., 1975) score of 17 or greater.

Eligibility criteria for MCI participants included: (1) DSM-5 (American Psychiatric Association, 2013) criteria for Mild Neurocognitive Disorder of any subtype as determined by a clinical assessment; (2) age of 60 years or older; (3) Montgomery-Asberg Depression Rating Scale (MADRS; Montgomery and Asberg, 1979) score of 10 or below; (4) has not met DSM-5 criteria for a Major Depressive Episode in the past 10 years or life time diagnosis of schizophrenia, bipolar disorder, obsessive compulsive disorder; (5) no diagnosis of alcohol or other substance use disorder within the past 12 months; (6) no significant neurological condition that would interfere with participation in the study; and (7) no use of a cognitive enhancer medication (acetylcholinesterase inhibitor or memantine) within the past 6 weeks. In addition to our eligibility criteria, participants with Mild Neurocognitive Disorder (or MCI) had to meet the clinical operational criteria of having a Montreal Cognitive Assessment (MoCA; Nasreddine et al., 2005) score ≤ 26 and a MMSE (Folstein et al., 1975) score ≥ 24.

Finally, eligibility criteria for HC participants included: (1) no lifetime diagnosis of a DSM-5 disorder except for simple/specific phobias; (2) age of 60 years or older; (3) MADRS score of 10 or below; (4) no significant neurological condition or unstable medical illness; and (5) no neuropsychological testing within the past 12 months.

### Procedure

#### Working Memory Task

The N-back task is a verbal working memory task that requires participants to determine whether the stimulus presented on a computer monitor is the same as, or different from the stimulus presented N trials back. During the task, black capital letters were presented on a computer monitor one at a time in a continuous sequence. Each letter was present on the screen for 250 ms, followed by a 3000 ms time frame to respond. Stimuli that were the same as the letter N trials back were labeled as Targets whereas stimuli that were different were labeled as Non-Targets. The 1- and 2-back conditions consisted of 15 min of continuously presented letters; the proportions of Target trials were 0.23 and 0.16, respectively (Rajji et al., 2017). N-back accuracy was assessed using d′, a sensitivity index based on the *z* scores of the hit rate and false alarm rate using the following formula:

$$d^{\prime }=z(H)-z(F)$$

Given that the *z* transform reaches infinity when percentages equal 0 or 100, we used a common adjustment whereby scores of 0% were assigned values of 1% and scores of 100% were assigned values of 99% (MacMillan and Creelman, 1991).

#### EEG Data Recording and Processing

EEG was recorded during the N-back task, using a 64-channel Synamps 2 EEG system with a 10-20 montage placement. Electrodes were referenced to an electrode posterior to Cz electrode. EEG signals were recorded using DC and a low pass filter of 100 Hz at 20-kHz sampling rate. The EEG data was processed offline using MATLAB (The MathWorks Inc., Natick, MA, USA) and EEGLAB toolbox. The data were processed in accordance with previously published methods (Rajji et al., 2017). In short, the data were down sampled to 1 kHz, filtered and segmented from −1400 ms to +3100 ms relative to the stimulus onset. Following this, an electrode-by-trials matrix composed of ones and zeros was created; a value of zero was assigned to an epoch that met any of the following criteria: (1) an amplitude larger than ± 150 μV; (2) a power spectrum that violated 1/f power law; (3) a standard deviation greater than three times the average of all trials; (4) corresponding column had more than 20% of rows (i.e., channels) coded as zeros. Additionally, an electrode was rejected if its corresponding row had more than 60% of columns (i.e., trials) coded as zeros. Epochs were then manually inspected to remove any trials containing irregularities and independent component analysis (ICA; EEGLAB toolbox; Infomax algorithm) was completed to identify and remove noise from the data including eye-blink traces and muscle artifacts. Finally, data were re-referenced to the average mastoid electrode.

### Analysis

#### Theta–Gamma Coupling (TGC)

Following published methods (Axmacher et al., 2010; Rajji et al., 2017), we first filtered the raw EEG signal for theta (4–7 Hz) and gamma (30–50 Hz) frequency ranges with second-order zero-phase shift. We then calculated the time series for gamma amplitude and theta phase using the Hilbert transform. Next, we created a concatenated signal of 5000 ± 150 ms using epochs for all trial types at each electrode. All epochs included the time from the stimulus onset to the time of response. This interval was selected for our analysis because prior studies suggest that it is the critical time during which the ordering of information is represented (Rajji et al., 2017). We created a 5000 ms concatenated signal because the modulation index (MI)—the measure of TGC—is dependent on the length of the signal. Thus, we chose a 5000-ms concatenated signal to ensure stability of MI.

To calculate MI, each phase of theta was binned into 18 20° intervals. The average amplitude of gamma at each theta bin was calculated and normalized, resulting in phase-amplitude distribution function. We then calculated the MI of gamma amplitude by theta phase by measuring the divergence of the observed amplitude distribution from a uniform distribution (Tort et al., 2010; Rajji et al., 2017):

MI=[(log(N)−H(P))]/log(N)

where *N* is the number of phase bins, log(*N*) represents the entropy of a uniform distribution, *P* is the relative amplitude distribution sorted according to phase bins, and *H*(*P*) is the entropy of the *P* distribution, which is calculated as follows:

$$H(P)=-\sum_{j=1}^{N}P(j)\text{log}\left[P(j)\right]$$

Based on this equation, higher coupling is associated with lower entropy *H*(*P*), which therefore results in a higher MI value.

MI for each electrode and an average of MI across the right and left frontal electrode (AF3/4, F7/8, F3/4, F1/2 and Fz), were calculated and used in the statistical analysis. Additionally, TGC was analyzed for all Target trials (i.e., correct and incorrect) as a weighted average based on the number of correct and incorrect responses. For additional analyses, TGC was also analyzed for all Non-Target trials (i.e., correct and incorrect) as a weighted average. These calculations were repeated for each condition.

#### Theta and Gamma Power

We also analyzed theta and gamma powers to assess TGC independently from these powers given that they represent different components of working memory. Once the signal was filtered into the appropriate bands as described earlier, theta and gamma power were calculated from the squared amplitude of the Hilbert transformed signal. A group average of induced spectral power for all frontal electrodes, weighted by response type (i.e., correct and incorrect) across Target trials was then calculated.

#### Statistical Analysis

All data were analyzed using the Statistical Program for Social Sciences (SPSS) version 23.0 (SPSS Inc., Chicago, IL, USA). One-way analysis of variance (ANOVA) and *χ*^2^ tests were used to evaluate differences between the three groups on demographic variables (i.e., age, sex, years of education, MMSE). The level of significance was set at *α* = 0.05. For all planned *post hoc* comparisons, the statistical threshold for significance was based on *p*-values using Bonferroni correction for multiple comparison.

All measures including TGC and accuracy were analyzed from processed EEG data. Furthermore, data were natural log (LN) transformed to approximate the normal distributional assumptions required by parametric statistical methods. The variables that were transformed included weighted MI for both Target and Non-Target trials, performance (*d′*) for both 1 and 2-back conditions, and gamma and theta power for 2-back target responses. We performed three separate one-way ANCOVAs, one with accuracy (LN *d′*), one with coupling (LN MI) for Target trials and the other with coupling for Non-Target trials as the dependent variables, and diagnostic group as a between-subject factor. Given that there were group differences in years of education, this variable was included as a covariate in our analyses. Additionally, paired-samples *t*-tests were conducted to compare TGC from Target trials with Non-Target trials for each diagnosis separately. To assess the relationship between N-back accuracy and TGC and its independence from the relationships between accuracy and theta or gamma power, a simultaneous linear regression was performed. Accuracy (LN *d′*) was the dependent variable and natural log transformed TGC, theta and gamma powers were the independent variables; this linear regression was performed with all participants.

## Results

Demographic information is presented in Table 1. The groups did not differ in age or sex and race distribution. There were significant differences in years of education, with fewer years of education in the AD group compared to both the MCI and HC groups. There were also significant differences in the MMSE scores, with significantly lower scores in the AD group followed by the MCI and finally HC groups.

**Table 1:** Demographics.

	HC (*N* = 31)	MCI (*N* = 34)	AD (*N* = 33)	*F* or *χ*^2^	*df*	*p*-value
Age (years)	73.5 ± 5.2	74.8 ± 5.9	76.5 ± 6.2	2.07	95	0.132
Sex (M:F)	14:17	16:18	13:20	0.43	2	0.807
Years of education	15.6 ± 2.4	15.2 ± 2.3	13.5 ± 3.7	5.32	95	**0.005**
Race:				4.32	2	0.115
Caucasian	18 (58%)	23 (68%)	27 (82%)		
Non-caucasian	13 (42%)	11 (32%)	6 (18%)		
MMSE	29.1 ± 1.6	27.6 ± 3.1	22.6 ± 3.2	80.34	95	**<0.001**

*Bold numbers indicate significance at α ≤ 0.05. Except for race, values are listed as mean ± SD. HC, Healthy controls; MCI, Mild Cognitive Impairment; AD, Alzheimer’s disease; MMSE, Mini Mental State Examination; d*f*, degrees of freedom; F, One-way ANOVA; χ^2^, Chi Square test*.

### Primary Analyses (2-Back Condition)

**Figure 1:**
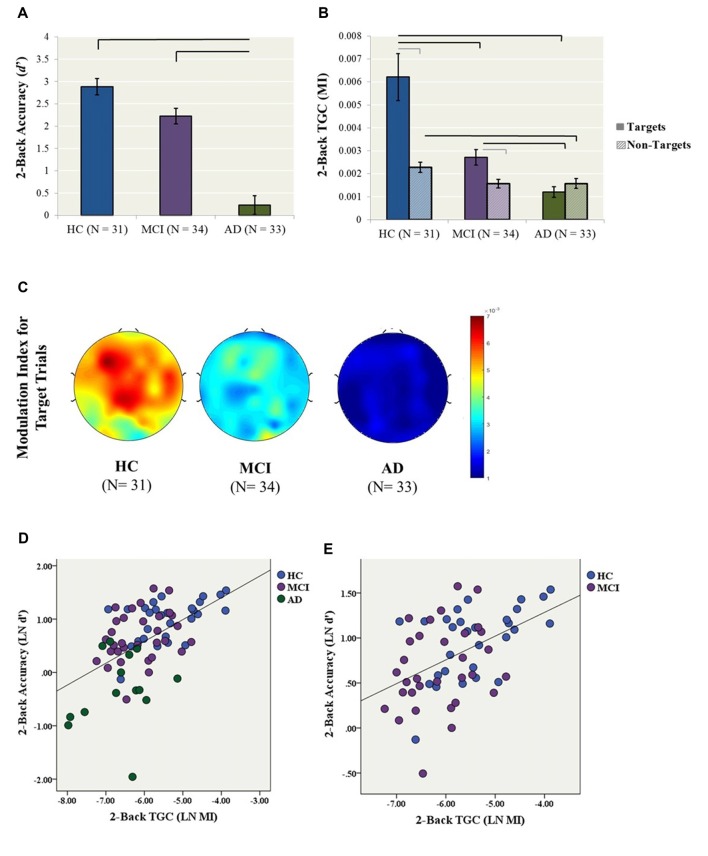
Primary analyses (2-back condition). **(A)** 2-back accuracy (d′) in healthy control (HC), mild cognitive impairment (MCI) and Alzheimer’s Dementia (AD) participants. Bars represent ± 1 standard error. The lines indicate statistically significant differences after Bonferroni adjustments. **(B)** Theta–gamma coupling (TGC) measured by Modulation Index (MI) in HC, MCI and AD participants for the 2-back condition. Coupling was measured from the frontal brain region (AF3, AF4, F5, F3, F1, FZ, F2, F4, F6) weighted for all Target and Non-Target trials. Black lines indicate statistically significant differences after Bonferroni adjustments for ANCOVAs for Target and Non-Target Trials, gray lines indicate significant differences for paired *t*-tests within diagnosis. Data for both Panels **(A,B)** were generated from the original data, while statistics were generated using log transformed data. **(C)** Topoplots illustrate 2-back TGC calculated as a weighted average from all Target trials **(D)** The relationship between TGC (MI) during all Target trials and 2-back accuracy across AD, MCI and HC participants (*R*^2^ linear = 0.286, *p* < 0.001). **(E)** The relationship between TGC (MI) during all Target trials and 2-back accuracy across only MCI and HC participants (*R*^2^ linear = 0.224, *p* < 0.001).

A one-way ANCOVA with diagnostic group (i.e., HC, MCI, AD) as the fixed factor, 2-back accuracy (i.e., LN *d′*) as the dependent variable, and years of education as a covariate showed a significant group effect on accuracy (*F*_(2,76)_ = 34.7, *p* < 0.001; Figure 1). In *post hoc* comparisons, the AD group performed significantly worse than both the HC (*p* < 0.001, Cohen’s *d* = 2.36) and MCI (*p* < 0.001, Cohen’s *d* = 1.74) groups following Bonferroni correction, while HC and MCI groups did not significantly differ (*p* = 0.057, Cohen’s *d* = 0.67; Figure 1A).

Similarly, a one-way ANCOVA with diagnostic group as the fixed factor, 2-back TGC for Target trials (i.e., LN MI) as the dependent variable, and years of education as a covariate revealed a significant group effect on TGC (*F*_(2,83)_ = 26.4, *p* < 0.001; Figure 1B). In *post hoc* tests, both the AD (*p* < 0.001, Cohen’s *d* = 1.92) and MCI (*p* = 0.003, Cohen’s *d* = 0.96) groups demonstrated significantly lower TGC compared to the HCs, as well as significantly lower coupling in AD compared to MCI (*p* < 0.001, Cohen’s *d* = 1.21). In contrast, the groups did not differ in 2-back gamma (*F*_(2,80)_ = 1.56, *p* = 0.22) or theta power (*F*_(2,83)_ = 0.78, *p* = 0.46).

In contrast to coupling from Target trials, the one-way ANCOVA with diagnostic group as the fixed factor, 2-back TGC for Non-Target trials (i.e., LN MI) as the dependent variable, and years of education as a covariate did not reveal a significant group effect (*F*_(2,87)_ = 3.012, *p* = 0.054). Additionally, paired-samples *t*-tests were conducted to compare the level of coupling from Target trials compared to Non-Target trials within each diagnosis. For the HC group, coupling was higher for Target trial compared to Non-Target trials, *t*_(28)_ = 4.72, *p* < 0.001. Similarly for the MCI group, coupling was higher for Target trial compared to Non-Target trials, *t*_(31)_ = 2.84, *p* = 0.008. In contrast, there was no difference in Target and Non-Target coupling for the AD group, *t*_(24)_ −1.37, *p* = 0.183 (Figure 1B).

Finally, a simultaneous linear regression was performed to determine whether TGC was associated with 2-back accuracy (LN *d′*) independent of gamma and theta power. A significant regression equation was found (*F*_(3,65)_ = 13.4, *p* < 0.001; *R*^2^ = 0.382). TGC was the most significant predictor of the model with a standardized beta = 0.693, *p* < 0.001, followed by gamma power with a standardized beta = 0.253, *p* = 0.038. In contrast, theta power was not a significant predictor of the model with standardized beta = −0.019, *p* = 0.858 (Figure 1D). Given that the AD participants performed significantly worse than all other groups, we performed another linear regression with only MCI and HC participants. The regression equation remained significant (*F*_(3,53)_ = 5.87, *p* = 0.002; *R*^2^ = 0.250). TGC was the only significant predictor of the model with a standardized beta = 0.450, *p* = 0.003 (Figure 1E).

### Secondary Analyses (1-Back Condition)

**Figure 2:**
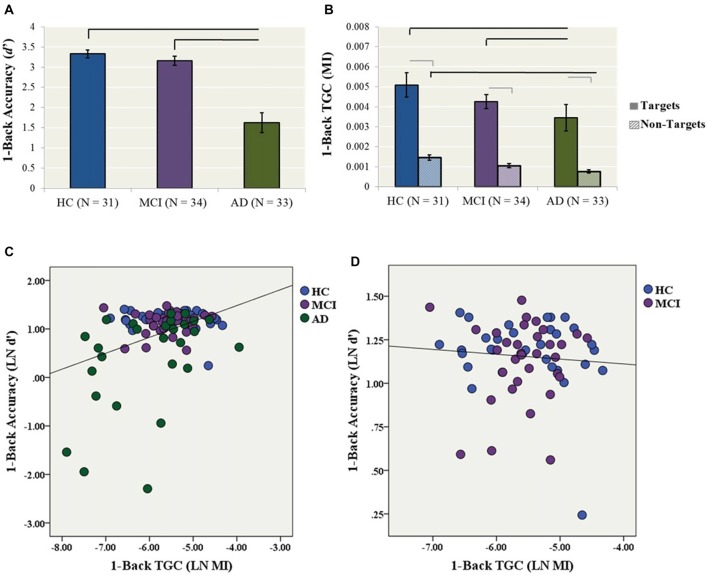
Secondary analyses (1-back condition). **(A)** 1-back accuracy (*d*′) in HC, MCI and AD participants. Bars represent ± 1 standard deviation. The lines indicate statistically significant differences after Bonferroni adjustments. **(B)** Theta–gamma coupling (TGC) measured by MI in HC, MCI and AD participants for the 1-back condition. Coupling was measured from the frontal brain region (AF3, AF4, F5, F3, F1, FZ, F2, F4, F6) weighted for all Target trials. Black lines indicate statistically significant differences after Bonferroni adjustments for ANCOVAs for Target and Non-Target Trials, gray lines indicate significant differences for paired *t*-tests within diagnosis. **(C)** The relationship between TGC (MI) during all Target trials and 1-back accuracy across AD, MCI and HC participants (*R*^2^ linear = 0.148, *p* < 0.001). **(D)** The relationship between TGC (MI) during all Target trials and 1-back accuracy across only MCI and HC participants (*R*^2^ linear = 0.006, *p* = 0.601).

A one-way ANCOVA with diagnostic group as the fixed factor, 1-back accuracy (i.e., LN *d′*) as the dependent variable, and years of education as a covariate showed a significant group effect on accuracy (*F*_(2,89)_ = 15.4, *p* < 0.001). In *post hoc* comparisons, the AD group performed significantly worse than both the HC (*p* < 0.001, Cohen’s *d* = 1.11) and MCI (*p* < 0.001, Cohen’s *d* = 1.04) groups, while HC and MCI groups did not differ (Cohen’s *d* = 0.27; Figure 2A).

Similarly, a one-way ANCOVA with diagnostic group as the fixed factor, 1-back TGC (i.e., MI) as the dependent variable and years of education as a covariate showed a significant group effect on coupling (*F*_(2,93)_ = 6.07, *p* = 0.003). In *post hoc* tests, the AD group had significantly lower TGC compared to the HC (*p* = 0.005, Cohen’s *d* = 0.72) and MCI (*p* = 0.008, Cohen’s *d* = 0.72) groups (Figure 2B).

The one-way ANCOVA with diagnostic group as the fixed factor, 1-back TGC for Non-Target trials as the dependent variable, and years of education as a covariate revealed a significant group effect (*F*_(2,93)_ = 10.5, *p* < 0.001; Figure 2B). In *post hoc* tests, the AD group had significantly lower TGC for Non-Target trials compared to the HC group (*p* < 0.001, Cohen’s *d* = 1.16), while there were no significant differences between AD and MCI (*p* = 0.065, Cohen’s *d* = 0.52) or MCI and HCs (*p* = 0.062, Cohen’s *d* = 0.68). Next, paired-samples *t*-tests were conducted to compare the level of coupling from 1-back Target trials compared to Non-Target trials within each diagnosis. For the HC group, coupling was higher for Target trials compared to Non-Target trials, *t*_(30)_ = 7.22, *p* < 0.001. Similarly for the MCI group, coupling was higher for Target trial compared to Non-Target trials, *t*_(33)_ = 10.24, *p* < 0.001. Coupling was also higher for Target trials compared to Non-Target trials for the AD group, *t*_(29)_ = 6.69, *p* < 0.001 (Figure 2B).

Finally, a simultaneous linear regression was performed to determine whether TGC was also associated with 1-back accuracy (LN *d′*) independent of gamma and theta power. A significant regression equation was found (*F*_(3,82)_ = 6.08, *p* = 0.001; *R*^2^ = 0.182). TGC was the only significant predictor of the model with a standardized beta = 0.375, *p* = 0.002. In contrast, gamma and theta power were not significant predictors of the model with standardized beta = −0.522, *p* = 0.603 and −0.404 *p* = 0.687, respectively (Figure 2C). The model no longer remained significant when only MCI and HC participants were included (Figure 2D).

## Discussion

In this study, we assessed TGC and its relationship to working memory performance in individuals with AD and MCI. We found that TGC was impaired in the AD and MCI groups compared to HCs and was the strongest predictor of working memory performance in these groups. More specifically, AD participants demonstrated the lowest level of TGC followed by MCI and then HC participants on the 2-back working memory task. In contrast, performance on the 2-back was only impaired in AD and not MCI participants compared to HCs. Taken together, these findings suggest that TGC is a specific measure of working memory function and that it is more sensitive to prefrontal cortical dysfunction than the behavioral assessment of working memory in individuals with MCI.

Few studies have evaluated EEG activity in AD and MCI using cross-frequency coupling. One recent study investigated a novel biomarker associated with phase-amplitude coupling estimates evoked during an auditory oddball task, as a means to differentiate amnestic MCI from HCs (Dimitriadis et al., 2015). The authors extracted coupling values across a number of frequency pairs, including TGC, and found significantly higher coupling values across multiple frequency pairs in 25 amnestic MCI participants compared to 15 HCs. In addition, using the various time-varying phase-amplitude coupling features and standard machine learning algorithms, a high degree of discrimination was found between MCI and HCs. The authors suggest that the increase in coupling represents a higher demand in the MCI group to synchronize attention and memory states. This contradictory finding to that of the current study may be due to a variety of differences between the two studies, including coupling analyses, participant inclusion criteria and the type of task administered during the EEG recording. A second study examined amplitude-amplitude coupling modulations during a resting state in HCs, patients with mild AD and a group with moderate AD (Fraga et al., 2013). The authors found changes in the modulation of several frequency bands including delta-theta and delta-beta with disease severity; however the relationship between theta and gamma was not assessed.

More commonly, researchers have approached the study of EEG activity in MCI and AD examining spectral and connectivity features of oscillations in relation to attention and memory functioning. One study examined the behavior of synchronization likelihood both at rest and during a memory task in AD and MCI. The authors reported no difference in theta or gamma synchronization likelihood during the memory task in AD or MCI when compared to older individuals with subjective memory complaints, although differences were found in other frequencies bands. Further, there was no association between synchronization likelihood and working memory scores during the task condition (Pijnenburg et al., 2004). A second study reported reduced theta coherence during the visual oddball paradigm in individuals with AD compared to HCs but did not report on any association between theta coherence and behavioral performance associated with the mental count of the target stimuli (Güntekin et al., 2008). A third study examined theta activity using a detection and N-back task in individuals who progressed from MCI to dementia compared to those with stable MCI and HCs. While the authors reported no difference in global theta power among the three groups, induced theta activity in the frontal region was reduced in the MCI group that progressed to dementia compared to the two other groups, regardless of task. This progressive MCI group also demonstrated lower accuracy compared to HCs (Deiber et al., 2009). Still, this study also did not assess for any relationship between behavioral performance and theta activity. Finally, one last study examined a ratio of theta/gamma relative power at peak frequency during rest EEG in individuals with MCI and found that theta/gamma ratio was negatively correlated with performance on a story recall test yet not on any other cognitive test (Moretti et al., 2009). The fact that EEG data was collected using an eyes closed rest paradigm and not during cognitive testing limits the ability to draw mechanistic conclusions regarding the role of theta and gamma oscillations in supporting cognitive function. Thus, our study advances the field by demonstrating an association between TGC and cognition *in vivo* and during working memory performance.

Additionally, our behavioral findings are consistent with previous findings in AD and MCI. Several studies have observed intact working memory performance on both 1- and 2-back accuracy in MCI (Döhnel et al., 2008; Guild et al., 2014; Migo et al., 2015). While these individuals often display deficits on tests of learning and episodic memory, they are generally able to maintain a limited memory set in mind for short time periods (Elias et al., 2000; Bäckman et al., 2001). By contrast, deficits in working memory performance are consistently observed in individuals with AD (Redondo et al., 2016).

The relatively intact working memory performance in MCI despite significantly impaired TGC suggests that there is a threshold for TGC above which accurate working memory performance can be maintained. Thus, while individuals with MCI experience less TGC than HCs, the amount of TGC they experience may be sufficient for accurate performance at this level of working memory load. As a corollary, the additional amount of TGC in HCs could represent a neurophysiologic reserve. Other neuroimaging (Clément and Belleville, 2010; Faraco et al., 2013; Migo et al., 2015) and electrophysiological (Pijnenburg et al., 2004; Dimitriadis et al., 2015) studies have also shown aberrant neural activity in the absence of observable cognitive deficits in individuals with MCI. These studies have typically found an over-activation of neural activity in response to cognitive tasks, interpreted as a compensatory mechanism. While our findings do not provide support for this theory, the results suggest that TGC may be a more sensitive measure than behavioral performance in detecting prefrontal cortical dysfunction in individuals with MCI.

These results should be interpreted in light of some limitations. First, the task we used to assess working memory—the N-back—does not allow for the evaluation of specific memory sub-processes, e.g., encoding vs. retrieval. Previous studies have demonstrated that individuals with MCI or AD show deficits within specific memory subprocesses (Belleville et al., 2007; van Geldorp et al., 2015). Furthermore, given the possibility that more than one cognitive domain is likely affected in these populations, the overall performance on the N-back may not represent a pure working memory function. However, previous studies in healthy and clinical populations have demonstrated that the N-back is a well suited assessment for the manipulation of information associated with working memory and its related TGC (Sun et al., 2015; Rajji et al., 2017). Future studies should validate these findings employing additional working memory tasks as well as in a more homogenous sample of single domain, amnestic MCI participants. Another limitation is our cross-sectional design: to confirm whether TGC can be enhanced to delay progression to AD, longitudinal studies are needed. Finally, we focused only on gamma and theta bands given the potential role of TGC in working memory. Future studies could investigate other forms of cross–frequency coupling including alpha-gamma coupling given previously identified alterations in several frequencies in AD, including alpha-band synchronization.

In conclusion, our results provide direct evidence of a relationship between working memory deficits and altered TGC in individuals with AD and MCI. This work advances our understanding of the mechanisms underlying cognitive deficits in these populations, with the ultimate goal of guiding the development of future therapeutic and preventative interventions for AD.

The raw data supporting the conclusions of this manuscript will be made available by the authors, without undue reservation, to any qualified researcher.

## Author Contributions

MSG analyzed the data and wrote the manuscript. MSG, RZ, ASMC, SK, ZG and TKR developed the analysis methods. BHM, TKR, DMB, CF, AF, LM, NH, SK and CRB developed the design of the parent study. ZJD, MSB and all other co-authors aided in interpretation of the results and editing of the manuscript.

## Conflict of Interest Statement

MSB’s work is supported in part by Brain and Behavior Young Investigator Grant. ZJD has received research and equipment in-kind support for an investigator-initiated study through Brainsway Inc., speaker support from Eli Lilly, Ontario Mental Health Foundation (OMHF), Canadian Institutes of Health Research (CIHR), the Brain and Behavior Research Foundation and the Temerty Family and Grant Family and through Centre for Addiction and Mental Health (CAMH) Foundation and the Campbell Institute. DMB’s work is supported by the CIHR, US National Institute of Health (NIH), Brain Canada, Temerty Family through the CAMH Foundation, Campbell Research Institute, in-kind equipment support for an investigator-initiated study from Brainsway Ltd., in-kind equipment support from Magventure for an investigator-initiated study and also receives medication supplies for an investigator-initiated trial from Invidior. CF receives research support sponsored by Roche pharmaceuticals and Vielight. AF currently receives grant support from the U.S. NIH, the CIHR, the Patient-Centered Outcomes Research Institute, Brain Canada and the Ontario Brain Institute. NH has received research support from Lundbeck, Axovant Sciences and Roche pharmaceuticals as well as consultation fees from Merck, Eli Lilly and Astellas Pharma. CRB has received consultation support from Boehringer Ingelheim, Lundbeck and Takeda Pharmaceutical Company as well as research support from Pfizer, Lundbeck and Takeda Pharmaceutical Company. BHM currently receives research support from Brain Canada, CIHR, the CAMH Foundation, the Patient-Centered Outcomes Research Institute (PCORI), the US NIH, Eli Lilly (medications for a NIH-funded clinical trial), Pfizer (medications for a NIH-funded clinical trial), Capital Solution Design LLC (software used in a study founded by CAMH Foundation), and HAPPYneuron (software used in a study founded by Brain Canada). He has also received research support from Bristol-Myers Squibb (medications for a NIH-funded clinical trial) and Pfizer/Wyeth (medications for a NIH-funded clinical trial). He directly own stocks of General Electric (less than $5000). TKR has received research support from Brain Canada, Brain and Behavior Research Foundation, Canada Foundation for Innovation, Canada Research Chair, CIHR, Ontario Ministry of Health and Long-Term Care, Ontario Ministry of Research and Innovation NIH and the W. Garfield Weston Foundation. MSG, SK, ZG, RZ, ASMC and LM report no financial relationships with commercial interests.
